# Sexual health in patients with malignant hematological disease: a Danish cross-sectional study

**DOI:** 10.1093/sexmed/qfae053

**Published:** 2024-09-13

**Authors:** Kristina Holmegaard Nørskov, Ida Schjoedt, Anders Tolver, Mary Jarden

**Affiliations:** Department of Haematology, Copenhagen University Hospital, Rigshospitalet, Copenhagen Ø 2100, Denmark; Department of Haematology, Copenhagen University Hospital, Rigshospitalet, Copenhagen Ø 2100, Denmark; Department of Mathematical Sciences, University of Copenhagen, Copenhagen N 2200, Denmark; Department of Haematology, Copenhagen University Hospital, Rigshospitalet, Copenhagen Ø 2100, Denmark; Department of Clinical Medicine, University of Copenhagen, Copenhagen N 2100, Denmark

**Keywords:** Sexual health, Hematologic malignancy, Sexual distress, Sexual quality of life, Erectile function, Sexual function

## Abstract

**Background:**

Patients who undergo treatment for hematologic malignancies may experience a decline in sexual health, alterations in sexual functioning, and reproductive capacity during survivorship.

**Aim:**

This study investigated the prevalence of sexual dysfunction and factors influencing sexual activity and functioning in patients with hematologic malignancies, to identify potential targets for interventions in clinical practice.

**Methods:**

This nationwide cross-sectional study included adult patients diagnosed with a hematologic malignant disease in Denmark in the period from January 20, 2013, to August 20, 2022. Eligible participants received electronic questionnaires through their officially assigned digital mailbox.

**Outcomes:**

Outcomes included the Female Sexual Function Index, International Index of Erectile Function, Female Sexual Distress Scale–Revised, European Organization for Research and Treatment of Cancer Quality of Life Questionnaire–Sexual Health, and European Organization for Research and Treatment of Cancer Quality of Life Questionnaire.

**Results:**

A total of 362 patients, on average 5.7 ± 3.4 years postdiagnosis, completed the questionnaires. Of these, 52.5% women and 73.2% men reported sexual dysfunction, with more women (40.9%) than men (34.1%) being sexually inactive. Across gender, this was significantly more prevalent in patients >65 years of age and in those with a low quality of life. In addition, for women a significant association with fatigue and sleep difficulties was observed. In total, 40.3% reported sexual-related personal distress, with the highest proportion among patients 40 to 65 years of age. Most patients (98.7%) with sexual dysfunction had not discussed sexual issues with their healthcare professional.

**Clinical implications:**

It is hoped that knowledge from this study will help healthcare professionals in clinical practice and encourage them to proactively address and discuss sexual health issues with their patients, irrespective of age.

**Strengths and Limitations:**

Sexually inactive participants may reduce the overall score of sexual function in the scoring of both the Female Sexual Function Index and International Index of Erectile Function. We therefore analyzed sexual function in a subgroup analysis in only those being sexually active to emphasize that level of dysfunction persists in sexually active participants.

**Conclusion:**

Patients report a high prevalence of sexual dysfunction, sexual distress, and gender-specific sexual symptoms following diagnosis and treatment of a malignant hematologic disease, impacting their quality of life.

Sexual Health in Patients With Hematologic Malignancies; NCT05222282; https://clinicaltrials.gov/study/NCT05222282.

## Introduction

Patients who undergo treatment for hematologic malignancies (HMs) may experience a decline in sexual health as one of the challenges during survivorship.^1–4^ Sexual dysfunction is characterized as a disruption in 1 or more elements of the sexual response cycle, and psychological factors such as body image, identity, sexual quality of life (QOL), and distress, all of which pose a threat to overall sexual well-being.^5^^,^^6^

Sexual dysfunction is prevalent in cancer patients and can result in diminished sexual desire and arousal, and difficulty achieving orgasm.^7^ In women, it comprises dyspareunia, vaginal dryness, insufficient lubrication, atrophy, and stenosis.^5–7^ Men experience sexual difficulties as erectile dysfunction (ED), ejaculatory dysfunction, hypogonadism, and sensitivity of the skin.^5^ In both genders, these complaints differ across cancer diagnosis, stage, and treatment type, and are influenced by individual factors like age. This supports the need to study men and women separately. Despite the negative impact of the disease and treatment on sexual function and fertility in cancer patients, there remains a lack of research, particularly in non–sex-specific cancer populations including HMs.^3^^,^^7^^,^^8^

Disease and treatment for HMs are associated with a high symptom burden and short- and long-term toxicities, which can impair sexual health and lead to alterations in sexual functioning and reproductive capacity.^1–4^ Symptoms, treatment side effects, and psychosocial stress can infringe upon QOL at all stages of the disease trajectory and have been associated with significant sexual dysfunction in both genders.^3^^,^^7^^,^^9–13^ Research on sexual health in this population is predominantly focused on fertility and sexual function in younger patients treated for Hodgkin lymphoma/non-Hodgkin lymphoma (hereafter referred to as lymphoma), or those who have undergone allogeneic hematopoietic stem cell transplantation (HSCT).^3^^,^^4^^,^^7^^,^^14–17^

In a longitudinal study, Olsson et al^18^^,^^19^ investigated sexual health in patients treated for HMs (N = 32) and found significant changes in sexuality and sexual function over time including decreased sexual desire and ability, negatively impacting QOL. Still, a recent systematic review (2020) exploring the prevalence of sexual problems among patients with HMs emphasized that the exact extent of the impact of diagnosis and treatment on sexual health remains to be answered.^3^ To provide appropriate counseling on sexual health to patients treated for HM diseases, healthcare professionals (HCPs) need to understand the epidemiology of sexual dysfunction and associated risk factors.

The aim of the study was to examine the sexual health of adult patients with HMs in Denmark and to investigate the prevalence of sexual dysfunction, understand the factors influencing sexual activity and functioning, and identify potential targets for intervention to improve sexual health.

The study was based on the following hypothesis: patients with HMs report impaired sexual function, and their sexual dysfunction is associated with high symptom burden and reduced QOL.

## Methods

This Danish nationwide exploratory cross-sectional study was conducted in accordance with the statement guidelines for reporting observational studies (STROBE [Strengthening the Reporting of Observational Studies in Epidemiology]).^20^ The study was conducted at the Department of Hematology, Copenhagen University Hospital, Rigshospitalet, Denmark.

### Participants

Participants *>*18 years of age were eligible if diagnosed with HMs, specifically acute myeloid leukemia/acute lymphatic leukemia (AL), chronic lymphatic leukemia/chronic myeloid leukemia (CL), multiple myeloma (MM), lymphoma, or myelodysplastic syndrome (MDS), with a time postdiagnosis of 6 months up to 10 years (January 20, 2013, to August 20, 2022).

### Recruitment

Eligible participants were identified through the National Patient Register, after which the Danish Health Data Authority extracted a representative sample from the full potential eligible participants (January 1, 2023). Eligible participants received the questionnaires in their officially assigned digital mailbox (e-Boks) with an information letter about the study and a direct link to a self-administered online questionnaire.

### Data collection

Participant and disease characteristics were collected from the participants. Patient-reported outcomes included the following questionnaires.

#### Primary outcome

Sexual function was assessed in female participants by the Female Sexual Function Index (FSFI), which provides scores on 6 domains: desire, arousal, lubrication, orgasm, satisfaction, and pain.^21^^,^^22^ In male participants, sexual function was assessed by the International Index of Erectile Function (IIEF-15) that provides scores on 5 domains: erectile function, orgasmic function, sexual desire, intercourse satisfaction, and overall satisfaction.^23^ Higher scores in both the FSFI and IIEF-15 indicate better functioning. A mean ED score below 26.0 (IIEF-15) for men and a mean sum score below 26.55 (FSFI) for women have been validated as clinical cutoff scores for the identification of ED and sexual dysfunction, respectively.^22^^,^^23^

#### Secondary outcomes

Sexual health was assessed by the European Organization for Research and Treatment of Cancer Quality of Life Questionnaire–Sexual Health (EORTC QLQ SH22).^24^

Sexual distress was measured by the Female Sexual Distress Scale–Revised (FSDS-R).^25^ The scale was originally developed for women and validated in men with excellent internal consistency and test-retest reliabilities.^26^ A sum score ≥11 was considered indicative of experiencing sexual distress and sexually related personal distress.^27^

Quality of life was assessed by the European Organization for Research and Treatment of Cancer Quality of Life Questionnaire (EORTC-C30).^28^

### Ethics

The Danish Data Protection Agency (P-2021-647) and the Danish Health Data Authority (FSEID-00006378), as well as Copenhagen University of Hospital’s ethics committee (58 097, 19-04-2017) and approved the study, and it adheres to the tenets of the Declaration of Helsinki. All participants were provided with written study information, informed that participation was voluntary, and assured that any identifying information would be anonymized and protected.

### Statistical analysis

Sample sizes were determined to allow comparison of diagnosis groups for each gender-specific primary outcome (sexual function).^29^^,^^30^ In female patients, assuming a difference of size 4 for the primary outcome (FSFI) between groups and an SD of 6.75 points for the primary outcome within each group, a 2-sample *t* test with a power of 80% would detect a significant difference at the .05 level if 46 patients were included in each diagnosis group. To account for an expected low response rate, we decided to increase this number to a group size of 184. In male patients, assuming a difference of size 7.27 for the primary outcome (IIEF-15) between groups and an SD of 10 points for the primary outcome within each group, a 2-sample *t* test with a power of 80% would detect a significant difference at the .05 level if 31 patients were included in each diagnosis group. To account for an expected low response rate, we decided to increase this number to a group size of 124.

Official scoring manuals, including guidelines for handling missing responses, were used for computation of subscale scores for questionnaires. Raw means and SDs were reported for all subscale scores for the total group and stratified by diagnosis. Mean subscale scores can be estimated with a precision (ie, length of 95% confidence intervals [CI]) of approximately 4 units for subgroups of size 30 with an SD of 30. Demographic, clinical, and dichotomized outcomes were summarized as numbers and percentages. Logistic regression was used to examine associations between the primary outcome (sexual function) and QOL, sleeping difficulties, fatigue, and sexual distress. A supplementary logistic regression model was used to identify risk factors of sexual dysfunction among the predictors age, time since diagnosis, marital status, treatment status (active, follow up, ended), and education level. Effect sizes were reported as odds ratios (OR) with 95% CIs. Secondary outcomes, consisting of subscale scores from questionnaires, were compared across diagnosis groups using an *F* test. Post hoc pairwise comparisons using Holm’s method were used to identify diagnosis groups with different mean scores. We analyzed sexual function (FSFI, IIEF-15) in a subgroup analysis in only those being sexually active to analyze if level of dysfunction persists in sexually active participants. Statistical analyses were conducted using R (R Foundation for Statistical Computing, version 4.4.1) and SPSS (IBM, version v29). *P* values below .05 were considered statistically significant. We emphasize the exploratory nature of the analyses based on secondary outcomes, due to the increased risk of a few false positive findings.

## Results

Of the 1540 invited participants, a total of 362 (23.5%) consented participation and completed the data collection. Nonresponders had a mean age of 67.7 years (range 18-98 years), with the majority being women (61.6%). The study population had a mean age of 64.1 years (range 18-91 years), with most being women (54.7%) and married or cohabitating (70.4%) (Table 1). AL (25.1%) and CL (24.0%) were the most frequent diagnoses. Among the younger (18-40 years of age) and middle-aged (40-65 years of age) participants, the majority had AL (82.9% and 31.4%, respectively), whereas older participants (>65 years of age) primarily had MM (22.9%) or CL (29.4%). The mean years postdiagnosis was 5.7 ± 3.4, with 36.5% being <4 years postdiagnosis. A total of 18.8% had received allogeneic HSCT, and 12.7% received autologous HSCT as a part of their treatment.

**Table 1:** Demographic and Clinical Characteristics.

Characteristic	Total Sample	Men (N = 164)	Female (N = 198)	Acute Leukemia	Chronic leukemia	Lymphoma (N = 54)	Myelodysplastic syndrome	Multiple myeloma
	(N = 362)			(N = 91)	(N = 87)		(N = 37)	(N = 67)
Gender (female), n (%)			198 (54.7)	54 (59.3)	49 (56.3)	30 (55.6)	20 (54.1)	34 (50.7)
Gender (men), n (%)		164 (45.3)		37 (40.7)	38 (43.7)	24 (44.4)	17 (45.9)	33 (49.3)
Age, mean (SD)	64.08 (16.0)	67.7 (14.0)	61.1 (16.8)	50.8 (19.1)	69.6 (10.8)	64.4 (16.4)	67.5 (16.4)	69.3 (9.4)
Range	19–92	19–92	19–88	19–84	29–87	31–87	22–88	44–92
Categories, n (%)								
18-39	35 (9.7)	8 (4.9)	27 (13.6)	29 (31.9)	1 (1.1)	2 (3.7)	3 (8.1)	0
40-65	105 (29.0)	39 (23.8)	66 (33.3)	33 (36.3)	22 (25.3)	23 (42.6)	5 (13.5)	18 (26.9)
65 or older	201 (55.5)	107 (65.2)	94 (47.5)	25 (27.5)	59 (67.8)	24 (44.4)	27 (73.0)	46 (68.7)
Unknown	21 (5.8)	10 (6.1)	11 (5.6)	4 (4.4)	5 (5.7)	5 (9.3)	2 (5.4)	3 (4.5)
Education, n (%)								
No high school degree	39 (10.8)	21 (12.8)	18 (9.1)	16 (17.6)	5 (5.7)	3 (5.6)	2 (5.4)	8 (11.9)
Vocational education	88 (24.3)	58 (35.4)	30 (15.2)	17 (18.7)	30 (34.5)	8 (14.8)	11 (29.7)	12 (17.9)
High school degree	19 (5.2)	5 (3)	14 (7.1)	8 (8.8)	0	4 (7.4)	3 (8.1)	3 (4.5)
2 year college	45 (12.4)	9 (5.5)	36 (18.2)	12 (13.2)	9 (10.3)	9 (16.7)	4 (10.8)	7 (10.4)
4 year college	104 (28.7)	37 (22.6)	37 (33.8)	24 (26.4)	23 (26.4)	20 (37.0)	10 (27.0)	23 (34.3)
Master’s degree or higher	64 (17.7)	32 (19.5)	32 (16.2)	14 (15.4)	18 (20.7)	10 (18.5)	7 (18.9)	13 (19.4)
Unknown	3 (0.9)	2 (0.6)	1 (0.5)	0	1 (1.1)	0	0	1 (1.5)
Marital status, n (%)								
Married or cohabitating	255 (70.4)	121 (73.8)	134 (67.7)	58 (63.7)	66 (75.9)	39 (72.2)	26 (70.3)	52 (77.6)
Single, separated, divorced, or widowed	102 (28.2)	40 (24.4)	62 (31.3)	31 (34.1)	19 (21.8)	15 (27.8)	11 (29.7)	14 (20.9)
Unknown	5 (1.4)	3 (1.8)	2 (1)	2 (2.2)	2 (2.3)	0	0	1 (1.5)
Occupation, n (%)								
Salaried employee	102 (28.2)	47 (28.7)	55 (27.8)	37 (40.7)	22 (25.3)	20 (37.0)	5 (13.5)	14 (20.9)
Unemployed	13 (3.6)	2 (14.2)	11 (5.6)	5 (5.5)	3 (3.4)	3 (5.6)	2 (5.4)	0
Retired employee	223 (61.6)	108 (65.9)	115 (58.1)	33 (36.3)	60 (69.0)	30 (55.6)	27 (73.0)	51 (76.1)
Sickness benefits	9 (2.8)	2 (1.2)	7 (3.5)	5 (5.5)	1 (1.1)	0	1 (2.7)	2 (3.0)
Undergoing education	12 (3.3)	4 (2.4)	8 (4)	9 (9.9)	0	1 (1.9)	2 (5.4)	0
Unknown	3 (0.6)	1 (0.6)	2 (0.5)	2 (2.2)	1 (1.1)	0	0	0
Diagnosis, n (%)								
Acute leukemia	91 (25.1)	37 (22.6)	54 (27.3)					
Chronic leukemia	87 (24.0)	38 (23.2)	49 (24.7)					
Lymphoma	54 (14.9)	24 (14.6)	30 (15.2)					
Myelodysplastic syndrome	37 (10.2)	17 (10.4)	20 (10.1)					
Multiple myeloma	67 (18.5)	33 (20.1)	34 (17.2)					
Unknown	26 (7.2)	15 (8.5)	11 (4.5)					
Time since diagnosis, n (%)								
< 1 year	9 (2.5)	4 (2.4)	5 (2.5)	2 (2.2)	2 (2.3)	0	1 (2.7)	4 (6.0)
1–3 years	123 (34.0)	58 (35.4)	65 (32.8)	36 (39.6)	22 (25.3)	20 (37.0)	10 (27.0)	25 (37.3)
4–7 years	86 (23.8)	37 (22.6)	49 (24.7)	18 (19.8)	30 (34.5)	5 (9.3)	12 (32.4)	17 (25.4)
> 7 years	122 (33.7)	53 (32.3)	69 (34.8)	29 (31.9)	27 (31.0)	25 (46.3)	10 (27.0)	19 (28.4)
Unknown	22 (6.1)	12 (7.3)	10 (5.1)	6 (6.6)	6 (6.9)	4 (7.4)	4 (10.8)	2 (3.0)
HSCT, donor source, n (%)								
Autologous	46 (12.7)	18 (39.1)	28 (60.9)	1 (1.5)	0	10 (21.7)	0	35 (76.1)
Allogeneic	68 (18.8)	26 (38.2)	42 (61.8)	49 (72.1)	2 (2.9)	0	13 (19.1)	3 (4.4)
Treatment status								
In treatment	91 (25.1)	45 (27.4)	46 (23.2)	13 (14.3)	23 (26.4)	2 (3.7)	13 (35.1)	34 (50.7)
In follow-up	203 (56.1)	91 (55.5)	112 (56.6)	48 (52.7)	57 (65.5)	31 (57.4)	22 (59.5)	31 (46.3)
End of treatment	63 (17.4)	27 (16.5)	36 (18.2)	30 (33.0)	5 (5.7)	21 (38.9)	2 (5.4)	1 (1.5)
Unknown	5 (1.4)	1 (0.6)	4 (2)	0	0	0	0	1 (1.5)

SD; standard deviation, HSCT; Hematopoetic Stem Cell Transplant.

### Sexual symptoms and sexual activity

**Figure 1:**
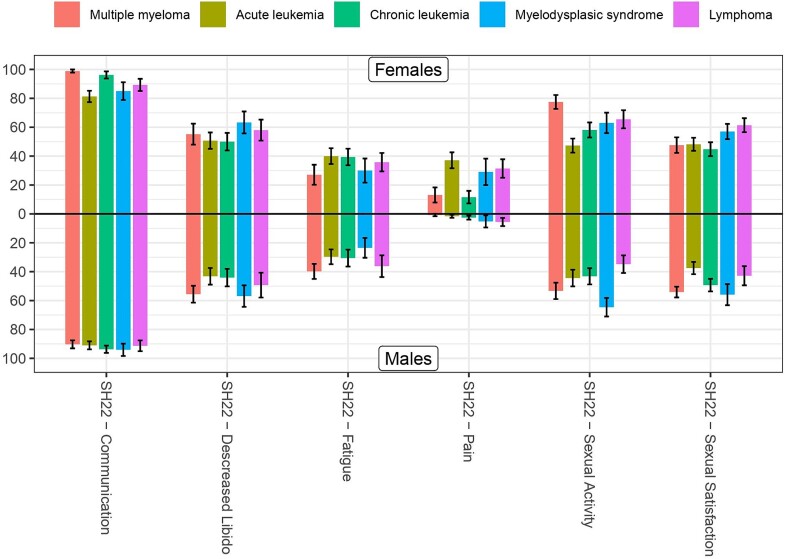
Specific sexual health domains across diagnosis and gender. SH22, European Organization for Research and Treatment of Cancer Quality of Life Questionnaire–Sexual Health.

We studied multiple QOL and sexual health domains (Table 2). High levels of fatigue were identified in both QOL and sexual health domains across diagnoses. Additionally, a high prevalence of sexual symptoms was reported in tandem with low sexual activity, decreased sexual satisfaction, decreased libido, vaginal dryness (women), and confidence in erection (men) (Table 2). Figure 1 illustrates specific sexual health domains across diagnosis and gender (all sexual health domains across diagnosis and gender are available in Tables S1 and S2). Levels of pain were predominantly reported by women with AL, lymphoma, and MDS (Figure 1). Overall, 37.8% (n = 137) reported not being sexually active within the last month, with a higher prevalence in women (40.9%) than in men (34.1%), and greater frequency in patients 65 years of age and older (45.3%). In women (n = 104) and men (n = 103) reporting being sexually active, the mean age in women was 56.1 ± 17.6 years (range 19-86 years) and in men was 64.2 ± 14.9 years (range 18-85 years). The majority of sexually active women were either middle-aged (39.4%) or older than 65 years of age (35.6%), and in men, most were >65years of age (59.2%).

**Table 2:** Patient-reported outcomes across diagnoses.

Variable	All participants (N = 362)		Acute leukemia (n = 91)		Chronic leukemia (n = 87)		Lymphoma (n = 54)		Myelodysplastic syndrome (n = 37)		Multiple myeloma (n = 67)		*P* value
	n	Mean ± SD	n	Mean ± SD	n	Mean ± SD	n	Mean ± SD	n	Mean ± SD	n	Mean ± SD	
**EORTC QLQ-C30**													
Global health (0-100)	360	69.7 ± 22	91	68.9 ± 23.3	87	71.7 ± 23.6	53	67.5 ± 20	37	67.6 ± 19.7	66	71.2 ± 20.9	NS
Physical functioning (0-100)	360	81.3 ± 19.8	91	82.6 ± 20.3	87	84.7 ± 19.2	53	79.9 ± 20.2	37	79.6 ± 21.9	66	77.6 ± 18.6	NS
Role functioning (0-100)	360	77.3 ± 26.7	91	77.7 ± 25.8	87	82.2 ± 25.9	53	74.8 ± 26.7	37	75.7 ± 28.2	66	74 ± 27.3	NS
Emotional functioning (0-100)	360	80.2 ± 22.3	91	80.2 ± 23.7	87	82.3 ± 22.5	53	78.3 ± 22.5	37	78.2 ± 18.4	66	82.1 ± 19.4	NS
Cognitive functioning (0-100)	360	80 ± 23.7	91	76.9 ± 28.1	87	84.7 ± 19.9	53	76.1 ± 26.3	37	81.5 ± 19.2	66	80.8 ± 20.7	NS
Social functioning (0-100)	360	80.9 ± 25.2	91	76.2 ± 28.1	87	88.1 ± 20.5	53	76.4 ± 27.2	37	79.7 ± 22.3	66	81.1 ± 23.2	.014
Fatigue (0-100	360	34.5 ± 26.8	91	35.4 ± 28.1	87	29.5 ± 28.4	53	40.4 ± 25.4	37	39.6 ± 26	66	31.5 ± 22.4	NS
Nausea and vomiting (0-100)	360	6.7 ± 15.1	91	6 ± 13.9	87	7.1 ± 17	53	4.7 ± 10.5	37	6.3 ± 12.6	66	6.6 ± 16.5	NS
Pain (0-100)	360	20.9 ± 26.5	91	20.1 ± 27.9	87	18.6 ± 28.4	53	24.8 ± 26.3	37	20.3 ± 23.3	66	23.5 ± 22.8	NS
Dyspnea (0-100)	360	17.3 ± 24.6	91	15.8 ± 24	87	14.6 ± 22.6	53	20.1 ± 24.8	37	16.2 ± 21.7	66	16.7 ± 25	NS
Insomnia (0-100)	360	28.7 ± 32	91	29.3 ± 34.4	87	28.4 ± 33.2	53	29.6 ± 29	37	31.5 ± 31.4	66	22.7 ± 26.3	NS
Appetite loss (0-100)	359	11.3 ± 22.9	91	9.2 ± 18.6	87	11.5 ± 27.3	52	10.3 ± 16.9	37	9.9 ± 22	66	10.6 ± 22.8	NS
Constipation (0-100)	360	13 ± 23.3	91	10.3 ± 20.9	87	14.6 ± 23.7	53	15.1 ± 25.8	37	10.8 ± 20.9	66	13.6 ± 23.4	NS
Diarrhea (0-100)	358	12.6 ± 22.9	91	9.2 ± 19.9	87	14.2 ± 26.2	51	10.5 ± 19.4	37	13.5 ± 21.5	66	15.7 ± 22	NS
Financial difficulties (0-100)	358	10.2 ± 22.2	91	15 ± 27.8	85	3.9 ± 14	53	15.1 ± 25.8	37	7.2 ± 19.5	66	10.6 ± 21.2	.008
**EORTC QLQ-C30 SH22**													
Sexual activity (0-100)	340	54.7 ± 34.8	86	46.1 ± 34.4	80	51.2 ± 34.8	52	48.1 ± 35.2	34	63.7 ± 27.7	64	66.1 ± 31.7	.002
Sexual satisfaction (0-100)	287	48.4 ± 26.8	81	43.6 ± 28.7	68	47.1 ± 26.7	38	52.1 ± 26.8	31	56.4 ± 24.9	50	51.3 ± 22.1	NS
Sexual pain (0-100)	279	13.6 ± 25.6	78	21.7 ± 32.8	64	7.1 ± 18.7	40	18.5 ± 25.5	29	15.9 ± 27.6	50	6.2 ± 17.4	.002
Decreased libido (0-100)	341	51.4 ± 37.6	87	47.5 ± 37.9	81	47.3 ± 38.7	50	54.0 ± 39.2	37	60.4 ± 32.2	62	55.4 ± 36.7	NS
Incontinence (0-100)	336	18.5 ± 30.9	86	14.3 ± 26.8	78	23.1 ± 33.7	50	18.7 ± 29.5	36	14.8 ± 30.3	63	16.4 ± 27.4	NS
Fatigue (0-100)	341	34.4 ± 35.8	87	35.6 ± 35.9	80	35.4 ± 36.9	50	36.0 ± 34.2	37	27 ± 33.2	63	33.3 ± 34.9	NS
Treatment (0-100)	277	44.5 ± 40.3	77	56.3 ± 42	59	29.4 ± 37.7	42	50.8 ± 38.4	30	35.6 ± 38.1	50	48.7 ± 38.8	.001
Communication with professionals (0-100)	342	91.2 ± 19.5	87	85.4 ± 24.2	81	95.1 ± 15.9	51	9.8 ± 20.3	37	89.2 ± 23.6	62	94.6 ± 12.4	.013
Partnership (0-100)	248	38.3 ± 38	70	33.3 ± 37.6	58	41.4 ± 41.6	38	46.5 ± 36.0	26	41 ± 36.9	39	34.2 ± 35.4	NS
Confidence erection (0-100)	157	55.2 ± 39	37	51.4 ± 39.7	37	55 ± 41.7	23	43.5 ± 34.0	17	62.7 ± 40.6	29	63.2 ± 34.9	NS
Body image (men (0-100)	158	28.1 ± 34.4	36	28.7 ± 34.9	37	28.8 ± 33.5	23	37.7 ± 40.6	17	19.6 ± 26.5	31	29 ± 33	NS
Body image (women (0-100)	187	27.8 ± 35.9	50	48.7 ± 40.5	46	14.5 ± 24	29	21.8 ± 34.8	20	41.7 ± 34	32	13.5 ± 27.9	<.001
Vaginal dryness (0-100)	102	50.7 ± 36.6	36	45.4 ± 36.6	24	48.6 ± 36.8	15	73.3 ± 38.2	9	63 ± 38.9	12	36.1 ± 17.2	.045
**FSDS-R**													
Global score (0-52)	302	13.2 ± 13.4	83	13.8 ± 13.7	69	14.5 ± 13.9	50	15.4 ± 14.3	32	12.5 ± 11.8	58	10.0 ± 10.9	NS
**FSFI (women**)													
Sum score (2-36)	133	15.3 ± 10.6	40	18.7 ± 11.2	33	14.8 ± 11.1	17	14.4 ± 7.9	14	10.6 ± 8	20	14.9 ± 10.7	NS
Desire (1.2-6)	174	2.4 ± 1.2	49	2.9 ± 1.4	41	2.4 ± 1.1	26	2.3 ± 1	20	2.1 ± 0.9	29	2 ± 1	.009
Arousal (0-6)	174	2.1 ± 2.1	49	2.9 ± 2.3	41	2.2 ± 2.1	26	1.8 ± 1.7	20	1.5 ± 1.8	29	1.7 ± 2.1	.045
Lubrication (0-6)	168	1.9 ± 2	46	2.6 ± 1.9	40	1.9 ± 2.1	26	1.5 ± 1.7	20	1.3 ± 1.8	27	1.4 ± 1.9	.037
Orgasm (0-6)	170	2.2 ± 2.4	49	3 ± 2.5	40	2 ± 2.5	24	1.7 ± 2	20	1.7 ± 2.1	28	2.1 ± 2.5	NS
Satisfaction (0.8-6)	141	3.4 ± 1.7	43	3.6 ± 1.9	33	3.4 ± 1.8	20	3.1 ± 1.3	14	3 ± 1.6	22	3.6 ± 1.4	NS
Pain (0-6)	167	2 ± 2.5	47	2.7 ± 2.5	39	2.2 ± 2.7	24	1.8 ± 2.2	20	0.4 ± 1.1	28	1.7 ± 2.6	.008
**IIEF-15 (men)**													
Sum score	139	33.1 ± 22.8	32	41.2 ± 22.7	29	28.8 ± 21.5	22	41.2 ± 23.4	15	18.6 ± 16.8	29	29 ± 20.7	.003
Erectile function (0-30)	148	12.1 ± 10.6	33	16 ± 10.9	34	10.1 ± 9.9	23	16.5 ± 11	15	5.6 ± 7.1	30	9.8 ± 9.8	.002
Orgasmic function (0-10)	153	4.8 ± 4.4	36	5.9 ± 4.3	34	3.8 ± 4.3	23	6 ± 4.1	15	2.7 ± 4.6	31	5.1 ± 4.6	NS
Sexual desire (1-10)	152	5.4 ± 2.4	36	5.8 ± 2.2	35	5.7 ± 2.3	23	6 ± 2.5	15	3.8 ± 2.1	29	5 ± 2.4	.031
Intercourse satisfaction (0-15)	148	4.7 ± 5.2	33	7.2 ± 5.2	33	3.4 ± 4.5	23	6.3 ± 5.5	15	1.5 ± 3.7	31	3.6 ± 4.6	<.001
Overall satisfaction (1-10)	147	5.6 ± 2.5	34	5.9 ± 2.3	32	5.2 ± 2.7	22	6.1 ± 2.7	15	5 ± 2.9	31	5.5 ± 2	NS

*P* values >.05 were considered NS.

Abbreviations: EORTC QLQ-C30, European Organization for Reseearch and Treatment of Cancer Quality of Life Questionnaire; EORTC QLQ-C30 SH22, European Organization for Research and Treatment of Cancer Quality of Life Questionnaire–Sexual Health; FSDS-R, Female Sexual Distress Scale–Revised; FSFI, Female Sexual Function Index; IIEF-15, International Index of Erectile Function; NS, nonsignificant.

### Sexual functioning

In women, the mean overall FSFI score was 15.3 ± 10.6, with 52.5% below the cutoff for sexual dysfunction (Table 2). However, among those who provided data on FSFI, this was 78.2%. The prevalence of sexual dysfunction ranged from 44.1% (MM) to 70% (MDS). In general, women scored low across all sexual function domains. Yet, women with AL reported better levels of lubrication (*P* = .037) and arousal (*P* = .045) across all diagnoses. Specifically, women with AL reported a higher level of sexual desire compared with patients with MM (*P* = .009) and lower levels of pain compared with patients with MDS (*P* = .008). In a subgroup analysis of sexually active women, the mean overall FSFI score was 21.2 ± 9.2, with 50.0% (n = 52) below the cutoff for sexual dysfunction (Table 3). The prevalence of sexual dysfunction ranged from 30.8% (MM) to 77.8% (MDS).

**Table 3:** Sexual function in sexually active patients across diagnoses.

Variable	All participants		Acute leukemia		Chronic leukemia		Lymphoma		Myelodysplastic syndrome		Multiple myeloma	
	n	Mean ± SD	n	Mean ± SD	n	Mean ± SD	n	Mean ± SD	n	Mean ± SD	n	Mean ± SD
**FSFI (women)**												
Sum score (2-36)	81	21.2 ± 9.2	32	21.8 ± 10.1	18	22.3 ± 9.7	10	18.8 ± 7	7	15.7 ± 7.7	9	24.8 ± 5.5
Desire (1.2-6)	94	2.9 ± 1.1	36	3.2 ± 1.3	20	3.1 ± 0.9	14	2.6 ± 0.9	9	2.5 ± 1	#	2.6 ± 0.6
Arousal (0-6)	93	3.5 ± 1.9	36	3.6 ± 2.1	20	3.6 ± 1.7	13	3.1 ± 1.5	9	2.7 ± 1.9	#	4 ± 1.8
Lubrication (0-6)	90	3.2 ± 1.7	35	3.3 ± 1.7	19	3.5 ± 1.7	13	2.7 ± 1.5	9	2.6 ± 2	9	3.5 ± 1.6
Orgasm (0-6)	91	3.4 ± 2.2	36	3.7 ± 2.2	19	3.7 ± 2.2	12	2.1 ± 1.9	9	2.5 ± 2.1	#	4.6 ± 1.9
Satisfaction (0.8-6)	85	4.2 ± 1.6	33	4.2 ± 1.8	18	4.4 ± 1.6	12	3.9 ± 1.1	7	4.1 ± 0.9	#	4.4 ± 1.6
Pain (0-6)	92	3.3 ± 2.4	36	3.3 ± 2.4	19	3.9 ± 2.6	13	3.2 ± 2.2	9	1 ± 1.6	#	4 ± 2.6
**IIEF-15 (men)**												
Sum score	93	43.8 ± 20.1	25	49.4 ± 18.2	21	35.8 ± 21.2	16	50.8 ± 19.4	6	33 ± 18.8	#	38.8 ± 19
Erectile function (0-30)	96	17.1 ± 9.9	25	20.1 ± 9	23	13.7 ± 10.1	16	21.2 ± 9.2	6	11.5 ± 8.4	#	14.5 ± 9.6
Orgasmic function (0-10)	99	7 ± 3.8	27	7.5 ± 3.3	23	5.5 ± 4.3	16	7.2 ± 3.5	6	6.7 ± 5.2	#	7.3 ± 3.9
Sexual desire (1-10)	98	6.2 ± 2	27	6.6 ± 1.9	23	6 ± 1.8	16	6.4 ± 2.5	6	4.5 ± 1.6	#	6.2 ± 1.8
Intercourse satisfaction (0-15)	96	7.1 ± 5	26	9.2 ± 4.1	22	5 ± 4.8	16	8.8 ± 4.8	6	3.8 ± 5.3	#	5.6 ± 4.6
Overall satisfaction (1-10)	97	6.2 ± 2.3	26	6.3 ± 2.2	22	5.5 ± 2.3	16	7.2 ± 2.1	6	6.5 ± 2.4	#	5.7 ± 2

Abbreviations: FSFI, Female Sexual Function Index; IIEF-15, International Index of Erectile Function.

In men, the mean IIEF ED score was 12.1 ± 10.6, with 73.2% (n = 120) below the cutoff for ED (Table 2). ED was more prevalent in patients 65 years of age and older (82.2%) and varied between diagnoses from 62.2% (AL) to 84.8% (MM). Specifically, ED was categorized as severe (65.8%), moderate (15.0%), mild to moderate (6.7%), and mild (12.5%). Severe ED was identified in 80.0% of the MDS patients with ED. In general, men scored lower in the sexual function domains. Across diagnosis, differences in erectile function (*P* = .002), sexual desire (*P* = .031), and intercourse satisfaction (*P* < .001) were identified, with a tendency of patients with MDS to be at particular risk. In a subgroup analysis of sexually active men, the mean ED score was 17.1 ± 9.9, with 66% (n = 68) below the cutoff for sexual dysfunction (Table 3). The prevalence of sexual dysfunction ranged from 50% (lymphoma) to 85%% (MM).

In general, sexual dysfunction was significantly more prevalent in patients older than 65 years of age in both men (*P* = .001) and women (*P* = .036) and increased significantly with lower QOL in both women (OR, 0.953; 95% CI, 0.927-0.977; *P* < .001) and men (OR, 0.971; 95% CI, 0.947-0.992; *P* = .011). Moreover, in women, sexual dysfunction increased significantly with higher levels of sleep difficulties (OR, 1.016; 95% CI, 1.001-1.033; *P* = .045) and fatigue (OR, 1.044; 95% CI, 1.023-1.070; *P* < .001).

### Sexual distress

The mean sum score for sexual distress was 13.2 ± 13.4, with 40.3% above the cutoff for experiencing sexually related personal distress. This varied across age groups, as 51.4% of patients 40 top 65 years of age reported sexual distress compared with patients 18 to 40 years of age (37.1%) and those older than 65 years of age (35.8%). However, sexual distress was equally distributed between genders (women 39.4%, men 41.5%) (*P* > .05). High sexual distress was significantly associated with sexual dysfunction in both women (FSFI sum; *P* = .003) and men (IIEF sum; *p* < .001.).

### Communication

In total, 98.7% of those with sexual dysfunction had not discussed sexual issues with their HCP. Specifically, in women with sexual dysfunction (n = 104), 97.1% had not communicated with HCPs about their sexual problems, and in men with ED (n = 120) this was 100%. Lack of communication with HCPs was equally distributed between diagnosis and gender (Figure 1).

## Discussion

### Discussion of results

This cross-sectional study assessed the sexual health of patients with hematologic malignancies in Denmark and explored factors associated with sexual activity and functioning, enabling identification of potential targets for interventions in clinical practice. We identified a high prevalence of sexual dysfunction and sexual distress in both genders, and several gender-specific sexual symptoms regardless of time of diagnosis, increasing with age. Sexual dysfunction was particularly prevalent in patients with MM and MDS, associated with low QOL and fatigue. The majority of those with sexual dysfunction had not communicated with their HCPs about their sexual problems. This is, to our knowledge, the first study to report prevalence data of sexual functioning in patients with CL, MM, and MDS.^3^ These findings emphasize the substantial symptom burden and long-term late effects of the disease and treatment for HMs, including impaired sexual health and altered sexual functioning.

Our results show that both women and men report a high prevalence of clinically relevant sexual dysfunction following the disease and treatment for HMs.

Women reported worse sexual function compared with women with solid organ cancer and noncancer, general population norms.^31^^,^^32^ Despite the overall prevalence of sexual problems in women, there were variations within the population, with a higher prevalence among those with MM and MDS. This difference can be partly attributed to age, as they were older compared with patients with AL or lymphoma. The evidence suggests that menopause in women is associated with significant changes, including vaginal atrophy, reduced vaginal lubrication, and decreased sensitivity in erogenous zones, and testosterone affecting libido.^33^ Moreover, urogenital atrophy in postmenopausal women can lead to problems not only in sexual functioning, but also in emotional well-being, interpersonal relationships, body image, and everyday activities such as cycling or prolonged sitting.^33^ Finally, sexual dysfunction in older women has been found to be strongly influenced by psychosocial factors and physical problems, including urinary incontinence.^33^ Thus, women in the general population are at increased risk of experiencing a wide range of sexual problems with advancing age. However, we found this risk to be further increased in women following the diagnosis and treatment for HMs, especially those with MM and MDS.

Men reported a high incidence of ED in our study, in particular impaired intercourse satisfaction. In the general population, ED and hypogonadism are the most common causes of sexual dysfunction in men, and their prevalence increases with age.^33^ With increasing age, testosterone levels naturally decline, which is associated with diminished libido. Sexual arousal and time to orgasm are lengthened, and erections require more physical stimulation to achieve and are reduced in frequency and durability.^33^ Regardless, men in our study reported lower sexual activity and reduced sexual function compared with the general population.^34^^,^^35^ These findings align with those of a systematic review that investigated sexual functioning in male survivors of lymphoma, in which low levels of sexual functioning and sexual satisfaction and reduced sexual activity were identified.^36^ As for men with ED, our findings suggest that ED is more prevalent in patients with MM and MDS compared with patients with lymphoma, AL, and CL. Specifically, men with MDS appear to be at high risk of experiencing severe ED.

Previous research has not found an association between intensity of chemotherapy and radiation and sexual functioning.^37^ Still, little is known about the associations between the type of diagnosis and sexual dysfunction. Hence, this relationship needs to be explored in larger-scale studies. It may be simply the type and length of treatment that matter, or it could be the curability of the disease or the remission state that influences QoL and sexual health. In this context, the HMs is probably cured in the AL patients that respond and participate in the study, whereas the MM and MDS patients most likely are still diseased and maybe even receiving treatment. Another important aspect is that sexual dysfunction is also prevalent in the general population.^31^^,^^32^ Due to the cross-sectional design in the present study, sexual function prior to diagnosis and treatment was not included. Thus, future robust longitudinal studies are needed to ultimately verify these findings on sexual dysfunction in patients with HMs.

Sexual dysfunction tends to increase with age, as the sexual response cycle can be influenced by age-related changes.^33^^,^^38^ Consistent with previous research, we found that older age was associated with decreased sexual activity and function.^4^^,^^15–17^^,^^37^ Yet, several studies have shown that older adults can maintain active and satisfying sexual lives in their later years, despite facing challenges related to their general physical health, psychological well-being, and relationship status.^34^^,^^35^^,^^38^^,^^39^ We found that a considerable proportion of older adults (>65 years) continued to be sexually active despite their increased risk of experiencing low sexual function. In this context, ageism is a challenge, as older adults may still encounter stereotypes that label them as asexual or less sexually active individuals. These stereotypes may cause personal embarrassment and stigma concerns for both patients and HCPs.^40^ We found that only a few of those with sexual dysfunction had communicated sexual problems to their HCPs. This highlights the importance of HCPs being proactive in initiating conversations about sexual health, including sexual distress with patients regardless of age during their treatment trajectory. Open and nonjudgmental communication may support patients in managing and addressing their sexual health concerns.

Sexual distress has been widely examined and is recognized as a diagnostic criterion for diagnosing sexual dysfunction.^41^ While older adults experience increased sexual difficulties with age, previous research has shown that only a minority experience significant sexual distress. In a cross-sectional study of 275 adults >65 years of age, 60% reported at least 1 sexual difficulty, but only 25% reported sexual distress.^42^ In our study, we found that older adults (>65 years of age), despite having a high prevalence of sexual dysfunction, experienced less sexual distress compared with middle-aged patients. Additionally, we identified the prevalence of sexual distress to be equally low in both older adults (>65 years of age) and younger adults (<40 years of age). In younger adults, this may be attributed to their generally lower levels of sexual dysfunction compared with middle-aged and older adults. However, in older adults, it may imply that a lifelong sexual history has a protective effect when experiencing increased sexual dysfunction across age, and that middle-aged adults are at high risk of experiencing distress as a consequence of their sexual dysfunction. However, these findings contradict a cross-sectional study examining sexual function in middle-aged breast cancer women, which found that despite a high level of sexual dysfunction, the participants reported normal levels of sexual distress.^43^ Importantly, these results underscore the challenges and vulnerabilities of patients with HMs, identifying them as a high-risk population for sexual-related distress.

Fatigue is one of the dominant symptoms following treatment of HMs.^44^^,^^45^ We identified high levels of fatigue across diagnoses, affecting both the QOL and sexual health domains. Additionally, fatigue and sleep difficulties were significantly associated with sexual dysfunction. This aligns with the findings of Behringer et al,^37^ who identified in a large cross-sectional study (n = 4160) that fatigue was associated with worsened sexual functioning in patients with lymphoma. Therefore, fatigue may partly account for the high prevalence of impaired sexual health in this population and should be seen as an important factor influencing sexual activity and function.

Time since diagnosis was in this study not associated with improved sexual function, suggesting that for long-term survivors sexual problems may not improve over time. However, this study was not intended to identify changes over time. Greaves et al^4^ found that sexual function improved with increasing time postdiagnosis and treatment in patients with lymphoma. Again, the age distribution within the different hematological diagnoses may in part explain these findings. Also, it is important to emphasize that the effect of time since diagnosis on sexual function may vary across diagnoses. Our finding of a significant association between sexual dysfunction and low QOL is fundamental in this context. If sexual problems persist years after their diagnosis in survivors of HMs, these findings suggest that long-term survivors may also experience impaired QOL. Evidence suggests that sexual health significantly impacts QOL and may reduce emotional distress and improve the psychosocial response to the diagnosis and complications following treatment.^9^^,^^10^^,^^46^ Hence, the initiation of targeted interventions aiming to improve sexual health may potentially improve QOL from time of diagnosis and treatment of HMs into survivorship.

### Discussion of methods

This study is the first to investigate sexual health in a Danish population of patients with HMs. The strength of our study is the nationwide and broad sample of patients with HMs, potentially increasing the representativeness and generalizability of our findings. A limitation is the cross-sectional design, which cannot establish a temporal association of exposure and outcome. Therefore, any temporal associations should be interpreted with caution. In addition, a low response rate (23.5%) may impact the validity of the findings. For the FSFI sum score, we observed an SD of approximately 10 in contrast to the value 6.75 used for the sample size calculation (see Table 2). This implies that the study is a little underpowered. Yet, for many subscale scores we observed an SD much less than 30. Consequently, the precision of reference values for subgroups obtained from our study is substantially smaller than what was mentioned in relation to the sample size considerations. Cultural factors were not collected from the participants. However, the Danish population is relatively homogeneous with respect to cultural diversity. In the scoring of both the FSFI and IIEF, “not being sexually active” may reduce the overall score of sexual function. We therefore analyzed sexual function (FSFI, IIEF) in a subgroup analysis in only those who were sexually active to emphasize that level of dysfunction persists in sexually active participants. The clinical cutoff for sexual dysfunction in women (FSFI <26.55) is independent of age. However, considering age-related menopause, hormonal, and metabolic changes, all of which represent risk factors for sexual dysfunction, particularly in older age, this may be questioned. In addition, it could inflate the diagnostic limit of sexual dysfunction, especially in women who are not sexually active. This also applies to sexual dysfunction in men (IIEF <26.0). Thus, the clinical cutoff could be lower than 25.66 and 26.0, respectively, and the findings should be interpreted with caution. Previous studies have pointed out that only the least burdened are likely to participate, which suggests a risk of this study to underestimate dysfunctional sexual health in this population.^47^ Despite these limitations, we believe that our study provides valuable insight into the understanding of sexual problems in survivors of HMs, shedding light on an overlooked aspect of their QoL.

### Clinical implications

Our findings highlight the importance of informing patients about the high prevalence of sexual dysfunction that can occur following their diagnosis and treatment for HMs. We recommend implementing systematic screening to identify patients in need of assistance and offering targeted interventions to address potential clinical and psychological issues associated with sexual symptoms. Additionally, our results provide valuable evidence supporting the development of multimodal interventions. A recently published guideline offers evidence-based recommendations for interventions, aimed to improve the common sexual issues faced by people with cancer.^48^ This guideline recognizes the need for variability in content delivery modality, dosage, and outcome measures. Regardless, in a clinical setting, systematic screening can identify patients in need of help with sexual health problems, including sexual function. HCPs can inquire whether patients would like to discuss these issues and whether they would like information or a referral for assistance. It is hoped that knowledge from this study will help HCPs in clinical practice and encourage them to proactively address and discuss sexual health issues with their patients.

## Conclusion

In summary, our study recognizes patients with HMs as a high-risk population prone to experience sexual dysfunction following diagnosis and treatment, specifically in patients with MM and MDS. We identified a high prevalence of sexual dysfunction in both genders and several gender-specific sexual symptoms significantly associated with sexual distress, age, fatigue, and low QOL. Importantly, the majority of those with sexual dysfunction had not communicated with HCPs about their sexual problems, underlining the significance of HCPs in clinical practice to address and discuss sexual health issues with this population. The findings may inform and guide HCPs in understanding the epidemiology of sexual dysfunction and associated risk factors in order to provide appropriate counseling on sexual health.

## Supplementary Material

Sublementary_Table_1_qfae053

Sublementary_Table_2_qfae053
